# Revisiting the human ‘interaction engine’: comparative approaches to social action coordination

**DOI:** 10.1098/rstb.2021.0092

**Published:** 2022-09-12

**Authors:** Raphaela Heesen, Marlen Fröhlich

**Affiliations:** ^1^ Department of Psychology, Durham University, Durham, UK; ^2^ Paleoanthropology, Institute for Archaeological Sciences, Senckenberg Center for Human Evolution and Paleoenvironment, University of Tübingen, Tübingen, Germany; ^3^ Department of Anthropology, University of Zurich, Zurich, Switzerland

**Keywords:** social interaction, primate communication, language, comparative research, joint action, evolution

## Abstract

The evolution of language was likely facilitated by a special predisposition for social interaction, involving a set of communicative and cognitive skills summarized as the ‘interaction engine'. This assemblage seems to emerge early in development, to be found universally across cultures, and to enable participation in sophisticated joint action through the addition of spoken language. Yet, new evidence on social action coordination and communication in nonhuman primates warrants an update of the interaction engine hypothesis, particularly with respect to the evolutionary origins of its specific ingredients. However, one enduring problem for comparative research results from a conceptual gulf between disciplines, rendering it difficult to test concepts derived from human interaction research in nonhuman animals. The goal of this theme issue is to make such concepts accessible for comparative research, to promote a fruitful interdisciplinary debate on social action coordination as a new arena of research, and to enable mutual fertilization between human and nonhuman interaction research. In consequence, we here consider relevant theoretical and empirical research within and beyond this theme issue to revisit the interaction engine's shared, convergently derived and uniquely derived ingredients preceding (or perhaps in the last case, succeeding) human language.

This article is part of the theme issue ‘Revisiting the human ‘interaction engine’: comparative approaches to social action coordination’.

## 1. The human interaction engine hypothesis

Human language is arguably the most powerful social tool that has ever evolved. The question of how and why language emerged in the human lineage has been of interest to various disciplines and is one of contemporary science's great puzzles. One particularly influential hypothesis suggests that the ecological niche of language use is face-to-face interaction [1–3]. Given that humans spend about half of their waking hours in close-range communicative interactions with lengthy and mutually engaging sequence structures, our species' interaction intensity seems to be unmatched in the animal kingdom [4].

Yet until today, it remains unclear which kinds of socio-cognitive abilities have paved the way for the emergence of language. An increasing number of researchers propose that our unique communication system evolved as an adaptation to a new problem: the coordination of collaborative action [1,5–7]. From this viewpoint, the advent of language was preceded by the evolution of unique interactional ethology, or a ‘cognition-for-interaction’ [4], enabling communication through a distinct set of cognitive and behavioural capacities, metaphorically described as the ‘interaction engine' [1,2]. This assemblage has been hypothesized to have played a key role in facilitating the evolution of modern human communication [6], and along with it the engagement in joint action (collaborative activities that involve shared intentions, commitments and goals ) [8–10].

Although an exhaustive list of the interaction engine's elements seems to be missing, most researchers would probably agree that they relate to four major components [1–3,11]: multimodality (here summarized as the ability to communicate through different sensory channels (visual, auditory, acoustic) and organs (e.g. hands or mouth), [6,12]), sequence organization (communicative acts that have a contingent relationship with the previous and following act, presuming a normative obligation to deliver appropriate responses at the next best occasion, [13,14]), turn-taking (rapid turns at talking with minimal response gaps between conversational turns, [15,16]) and intentionality (the ability to communicate and respond to intentions not behaviours, [1,3]). As such, the interaction engine's ingredients are not some distinct brain modules but describe distinct principles of human interaction that are universally observed across the world's cultures [1], and for which scientific enquiry of their biological origins is warranted. These interaction principles broadly encompass structural features at the interaction level (e.g. turn-taking, communicative repair, sequence organization), made possible through a set of cognitive abilities at the individual level (e.g. theory of mind, communicative capacities to represent others' minds and to recognize intentions) [17].

Although language clearly transformed our sociality in unique ways [18], the interaction engine hypothesis states that it is the interaction engine that made it possible in the first place, not the reverse; as Levinson notes, language seems to be the *explicandum*, not the *explicans* [1, p. 42]. This assumption is based on the observation that the interaction engine is largely independent from language—like when interlocutors communicate without sharing the same language (e.g. tourists communicating with locals, [1]), deaf children who develop unique home signing systems as a consequence of growing up in families of hearing parents and without access to conventional sign languages [19], or when language use is prevented, as in some experimental paradigms [20].

Thus, instead of focusing on language itself, our theme issue prioritizes the question of how the interaction engine evolved ('came together') and which of the various elements made language possible. Particularly, this collection of papers seeks to identify the evolutionary origins of the interaction engine's various components. As for other complex human traits such as culture, this may be achieved by disentangling ingredients that are uniquely derived (only present in humans) from those that are phylogenetically inherited (shared with closely related primate relatives) or convergently derived (shared with more distantly related species owing to analogous environmental and social pressures).

Studies from recent years have already compiled an interesting set of evidence, warranting an update of the interaction engine hypothesis. Some species of nonhuman primates were shown to engage in communicative turn-taking [11,21,22], exhibit communication that is apparently organized in sequences, or ‘adjacency pairs' [21,23,24], engage in latent forms of self-initiated, communicative repair [25,26], and communicate and behave in ways suggestive of joint commitment [23,27–30]. But if many of our socio-cognitive abilities are not uniquely derived, what made language evolution possible? Which are the key capacities that are clearly human-unique and favoured language evolution in our lineage? And how meaningful are such cross-species comparisons, given that they are mostly based on a top-down approach in which we always face the risk that similar behaviours observed in other animals may involve underlying cognitive skills different from those presumed in humans?

To get better answers to these questions, we need synthesis and interdisciplinary dialogue. Most of the primate (including human) research papers on social action coordination are scattered and have not been brought into direct connection, which hampers scientific progress on the links between interaction, coordination and communication/language. Indeed, human interaction falls into an ‘interdisciplinary no-man's land' [1 p. 39]. It has been under study among many disparate fields, including evolutionary biology, ethology, linguistics, psychology and sociology. Immense efforts have already been made to bridge disciplinary divides in the research area of human social interaction itself [17,31], yet the concepts originating from this still remain largely inaccessible for the study in nonhuman animals (i.e. they are hard to operationalize empirically). Only recently researchers have started to bridge divides in extending human joint action concepts to nonhuman animals [15,21,22,29,30,32,33]. Unfortunately, research on social interaction often targets specialist audiences, creating a gulf between disciplines. Advances in the form of individual contributions within certain disciplines are often not accessible to others (e.g. owing to discipline-related jargon and institutionally restricted journal subscriptions). Because of the way academic disciplines and departments are organized, the primary goal of studying *social interaction* as a comparative subject has fallen through the cracks.

This theme issue is a first step in a new direction, with the broader aim of making the interaction engine hypothesis accessible for human *and* nonhuman scientific enquiry—so that empirical data can inform the theory, rather than the reverse [34]. By doing so, we revisit long-standing questions on the comparable features and principles of social interactions in human and nonhuman animals, and discuss how this research can inform the evolution of human communication. We hope that a direct interdisciplinary debate about human interaction concepts and their operationalization across disciplines will prove highly productive, not only in terms of the comparative study of the interaction engine components themselves, but also in terms of possibilities for the wider fields of comparative cognition. Even though the contributions mainly focus on primates, this theme issue seeks to identify methods and empirical coding schemes suitable for cross-species comparisons in general. Thus, it will hopefully also speak to comparative researchers studying social interactions beyond the primate realm.

## 2. Revisiting the interaction engine: nearly two decades later

Before introducing the various contributions of our theme issue, we must make an important clarification. This theme issue is by no means an attempt to claim that human conversation does not differ in marked ways from animal communication; by contrast, we acknowledge the immense transformative power of language in ramping up human intersubjectivity at its core [18]. Instead, we seek to gather evidence of similarities and differences of those qualities that might have acted as stepping stones to language. This amounts to a synthetic view not found elsewhere, focusing on the question: which key interactional capacities had to be in place for language to evolve? Rather than collecting independent papers over decades, we believe the answers can be found through a direct meeting of views and minds, which we hope to have achieved with this theme issue.

To revisit the influential idea that modern human communication was spurred by a unique interactional ethology [1,2,4,35], our theme issue combines contributions from experts who were among the first to put forward such theories (e.g. [1,5,7]), along with colleagues from succeeding research generations whose empirical and theoretical works re-evaluate, and thereby corroborate but also contest, initial claims. The theme issue is divided into four sections. In the first section (a), the idea of the interaction engine is addressed more broadly, focusing on the overall assemblage of socio-communicative capacities. The second to fourth sections each deal with closely related concepts, including contributions on different aspects of the interaction engine: (b) multimodal and face-to-face communication, (c) sequence organization, repair and joint commitment, and (d) intentionality.

### (a) The interaction engine as assemblage

The human interaction engine was originally introduced as the interactional base of language, composed of different layers, each having different phylogenetic and ontogenetic origins [1]. In the first contribution of this theme issue, Stephen Levinson [3] recapitulates his influential theory from nearly two decades ago, summarizing four fundamental components of this multi-layered system, comprising multimodality, turn-taking, sequential contingency and intention recognition. Arguing that the first three features have clear precursors in the communicative behaviour of other primates, for the fourth and least understood component, intention recognition, he explores a new evolutionary route: cuteness selection**.** Levinson's central point is that the generalization of empathic tendencies and prosociality within the maternal relationship to the group level could have driven the generalized ‘theory of mind' required for modern human communication.

Looking beyond phylogenetic origins, Judith Burkart and her colleagues [11] discuss the role of convergent evolution in the emergence of specific interaction engine features. They point to a double legacy in humans, with a powerful cognitive apparatus inherited from ape-like ancestors on the one hand, and novel motivational components added as result of convergent evolution on the other (e.g. shared levels of prosociality linked to the cooperative breeding systems of callitrichid monkeys and humans). According to the authors, it is the *combination* between phylogenetic and convergent components that must have shaped our unique set of socio-cognitive skills [11]. Nonetheless, it requires further scientific scrutiny to understand which elements were shaped by environments or social systems like cooperative breeding, and which were phylogenetically inherited from our ape-like ancestors.

### (b) Multimodal and face-to-face communication

Like the communication systems of many primates, human language is inherently multimodal, comprising various communication organs and sensory modalities [6]. The articulators deployed to communicate purposefully can be flexibly changed in both humans and nonhuman great apes, e.g. information transfer can shift from the mouth to the hands or other parts of the body, and reverse [36–40]. To illuminate the interplay of manual gestures, vocalizations and other communicative modalities in face-to-face interaction, it is important to study communication holistically, to ultimately assess both the flexibility of information transfer and the role each communication organ plays across different primate species [6,41,42]. The following contributions stress the relevance of such a multimodal approach by demonstrating that humans communicate not only via speech but also via nonconventional, nonverbal signals.

Judith Holler [12] provides a rich overview of human multimodal communication and discusses the central but often overlooked contribution of visual bodily signals in human every-day communication and the coordination of minds. She demonstrates that nonverbal signals are fundamentally integrated into human communication and play a pivotal role in pragmatics. This role becomes particularly clear through the focus on *non-iconic* manual signals, *bodily* signals, and *combinations* of these. This contribution emphasizes once again that the native environment for human communication is face-to-face interaction, and natural selection must have directly operated in this environment. Crucially, she articulates both similarities (the flexible use of multimodal and multicomponent signals) as well as differences in the way humans communicate compared with other apes (humans' ability to use bodily signals to achieve mutual understanding and to refer to it in the future).

Nonverbal communication is also particularly pervasive in early human development, stressed by the study by Gideon Salter & Malinda Carpenter [43]. They analysed a variety of observational and experimental data on communication in 6–12-month-old human infants, investigating communication during face-to-face mother–infant interactions. For the first time they document the processes leading to the emergence of two conventional gestures, showing and giving, which are among the earliest means by which infants create events of joint attention with social partners. Focusing on emergent pre-conventional, or ‘incipient', forms of behaviours that lead to conventional forms of gestures, they argue that these signals are the product of a series of gradual cognitive and motoric developments in the context of repeated social interactions. Their findings suggest that socio-interactional experiences with caregiver-assisted and -initiated acts of joint attention are the core niche in which conventional signal use emerges (in contrast to great apes, who presumably do not engage in triadic interactions at this level, [44,45]).

### (c) Sequence organization, communicative repair and joint commitment

In addition to its multimodal character, human communication is also inherently cooperative, evident both in structure and underlying prosocial motivations [1,46,47]. Conversation is organized in sequences, where one produced action leads to a predictable next response, such as in the case of greetings and question–answer pairs; cooperativeness is reflected in the way by which interlocutors respect sequencing rules. Openings and closings of interactions in humans, for instance, are based on such normative, ordered series of sequences [48,49]. Normativity also plays a role in communicative repair, where, through a misunderstanding in hearing or content of an utterance, the orderly sequence of conversation is disturbed; sequence organization thus provides the resource for recognizing breakdowns of intersubjectivity [50]. Such breakdowns are fixed by signallers who spontaneously correct or repeat a previous utterance following a repair cue by a receiver (other-initiated repair), or by their own initiative (self-initiated repair) [51–53].

Lorenza Mondada & Adrien Meguerditchian [24] demonstrate that sequence organization appears to be present also in the interactions of baboons (*Papio anubis*), thus rejecting the claim that only human interaction is characterized by orderly sequences of action. They apply a multimodal conversation-analytical approach to the study of baboon communication, finding evidence for sequentiality in interactional openings, where baboons' close monitoring of and reactions to adjacent turns mutually shape the interaction moment-by-moment. They conclude that baboons might have expectations of the kinds of reactions that should follow their communicative moves. Thus, the authors argue that the notion of sequentiality can be extended to the study of how nonhuman participants come to engage in joint activities, offering a scheme for comparing the interaction structures among human and nonhuman primates (see also [23,30]).

Raphaela Heesen *et al.* [25] further extend this view by dissecting communicative repair and thus preparing it for comparative research. They recognize that communicative repair in humans relies on four different empirical components (self-correction, repetition, elaboration and other-initiated repair), each presumably varying in the required cognitive skills, and that some of these are present in nonhuman primates. The authors point out that other-initiated repair, the form where signallers repair a previous utterance following a recipient's cue of misunderstanding, might only occur in humans, possibly because it requires theory of mind and conventional language [18]. In recognizing that human repair has precursors in other primates, this primer delivers a relevant comparative scheme for future work on the evolution of repair.

Adopting Heesen *et al.*'s notion of persistence and elaboration as cognitive building blocks of repair, Marlen Fröhlich & Carel van Schaik [26] present findings of gestural redoings from a comprehensive sample of wild and captive orang-utans of two species known to differ in social tolerance and sociability. Specifically, they address the question of whether the environment and social setting can foster self-initiated gestural redoings after communicative failure. In scrutinizing repetition and elaboration in gesture use, the authors find that the research setting predicts elaborated gestural redoings in Bornean orangutans (the less socially tolerant species in the wild), insofar as elaboration is more frequent in captive compared with wild individuals and more successful in captivity. This confirms the idea that both the immediate and developmental environments shape a species' interactional ethology, emphasizing how social and environmental factors can trigger the emergence of certain interaction engine capacities (see also [11]).

Normativity also invokes social accountability [18], or joint commitment, as the feeling of mutual obligation that binds participants to a joint action [8,10,54,55]. The principle of joint commitment is tightly linked to a normative understanding of how one is to act when engaging socially. Violations of social norms like breaching of turn-taking rules, or suddenly departing midway during an interaction without explanation, can invoke rebuke, which participants feel obligated to avoid [18,54]. Although conventional language certainly *facilitates* the regulation of joint commitment in many ways (e.g. through predetermination of commitments prior to the interaction [56]), it is not always *obligatory*. Adrian Bangerter *et al*. [8] argue that the feeling of mutual obligation intrinsic to joint commitment (the *product*) is not always formulated explicitly, but can emerge from a gradual, coordinated *process* of (not necessarily conventional) signal exchanges during joint action. Given that experts, including philosophers, agree that neither promises nor agreements are needed to establish a joint commitment, it becomes plausible that nonhuman primates and possibly other species might engage in joint commitments, something for which there is now some preliminary evidence in bonobos and chimpanzees [23,27,28]. The authors show that commitments always vary in strength, are affected by prior actions, depend on stacking and persistence, need to be reinstated after interruptions, and go beyond spoken language. These aspects bring about new perspectives for assessing joint commitment in the spontaneous joint activities of nonhuman animal species.

Adopting the framework of joint commitment-as-process, Federico Rossano *et al.* [57] present observations of spontaneous communication in young children aged 2 and 4 years when engaging in social actions with peers in preschools. The authors demonstrate how children enter into and exit from social actions, and compare their results with recent work on great apes' social interactions [23]. Their central conclusion is that although both human children and apes communicate when entering and exiting from interactions, in contrast to apes, young children engage in a variety of fast-paced interactions with multiple partners. The authors stress that such data, when based on consistent coding methods, are particularly suitable for valid ecological comparisons of social action coordination between species.

### (d) Intentionality

Second- or higher-order intentions (i.e. aiming to influence the recipient's knowledge state rather than their behaviour) have been discussed as potentially unique features of human communication (58,59; but see [60] for potential evidence of second-order intentionality in chimpanzees). It is evident that humans are extraordinary skillful in expressing and recognizing intentions. As Levinson [3] notes, it does not take much for you to understand that you still have breakfast on your face when I rub my chin with an indicative look during breakfast. Humans are intention-readers and human communication is in itself a context-dependent process of social inference [9]. Humans not only communicate ostensively, via ‘Gricean intentions' (speakers wanting to have their intentions recognized), but also infer intentions from others' utterances against the background of pragmatic information on context, previous interactions, and relationships. Humans constantly establish and mutually refer to common ground—a platform of common beliefs and knowledge that stacks up through repeated interaction and builds the foundation against which signals and actions are being interpreted [9,61,62]. Whether ostensive–inferential communication is uniquely derived in our own species or shared with other hominids remains debatable, and is a topic addressed by various contributions within and beyond this issue [34,59,63].

A novel approach of studying pragmatic reasoning abilities in the multimodal communication of nonhuman great apes is presented by Manuel Bohn *et al*. [64]. The authors analysed signal combinations in chimpanzees through a computational modelling perspective and find that the difference in the communication between them and humans appears not to lie in the kinds of signals being used (e.g. pointing gestures) but in *how* the signals are used (i.e. whether information about social relationship and context is provided). Their model could explain the reported differences in apes' and humans' comprehension of pointing, insofar as pointing itself might be too ambiguous unless enriched with pragmatic information. It raises the critical questions of whether great apes' signal comprehension in pointing experiments would be more likely to resemble that of humans if conditions were more appropriate (signals enriched with pragmatic meaning).

Whether intention recognition also plays a constraining role in great ape cooperation is addressed by Alicia Melis & Federico Rossano [58]. The authors specifically discuss the cross-modal communicative strategies und underlying degree of intentionality during experimental cooperative stag hunt scenarios—a possible evolutionary niche of the interactional base for language [44]. They propose that great apes' communicative performance in such cooperation settings might be constrained owing to their limited capacity in comprehending helpful intent, insofar as signals are mainly understood as imperative acts (signallers wanting something) rather than as helpful cues (signallers wanting to share helpful information). What remains unclear is whether this constraint is due to an inability to comprehend helpful intent or due to setting-related factors, such as differences in proximal versus distal setups, kin and dominance relationships, or methodological limitations.

Yet, despite the relevance of ostensive–inferential communication in human evolution [3,44], it remains among one of the most obscure features of the interaction engine. This is partly due to the inherent difficulty of operationalizing such concepts for comparative research. By observation alone we will never have access to a social agent's internal cognitive processes, and without the presence of language in other species we directly depend on external behavioural indications that can at best be *suggestive* of higher-order intentions or the lack thereof. In her opinion piece, Christine Sievers [34] addresses this issue by illustrating how nonhuman species are denied an ostensive communicative capacity based on *a priori* theoretical exclusion. Sievers argues that ostensive communication requires theoretical re-analysis to enable comparative investigation and that the theory should be constructed based on empirical evidence rather than conceptual presumptions. She advocates a better interactional understanding of how nonverbal communication naturally unfolds in animal communication, thereby considering the individuals' shared interactional experience.

In a final opinion piece, Michael Tomasello [44] attempts to reconcile the idea of human social action being unique in many ways with the accumulating evidence on potentially shared capacities of the human interaction engine. In reviewing recent findings, he defends the hypothesis that only humans coordinate joint attention recursively—with the mutual understanding that they are doing so together—and engage in mutually obligating joint goals and commitments via acts of intentional communication. Similar to other scholars [18], he notes that conventional language is essential for the effective coordination of joint commitments in humans. Tomasello concludes that apes have not evolved shared intentionality because they have not undergone a similar pressure for collaborative foraging, thus pointing out potential species-specific adaptations in humans that might have favoured language evolution.

## 3. Do nonhuman species exhibit components of the interaction engine?

How does the new evidence of primates' communication and coordination capacities affect our understanding of the human interaction engine? Providing a clear answer to the question of whether nonhuman species exhibit certain interaction engine components is often difficult, partly because of the contradictory findings regarding coordination skills in naturalistic versus experimental settings. On the one hand, experimental evidence for the ability of producing and especially comprehending communicative signals that would aid the coordination in cooperation paradigms appears to be fairly limited in nonhuman species, such as chimpanzees [44]. However, recipient affordances such as physical distance and familiarity might affect the coordination process in these artificial scenarios [58], as they have a major impact on effective communication. Thus, many field researchers who have witnessed spontaneous cooperative interactions in a more natural setting may argue that it is inappropriate, or at best premature, to assume that ‘communicating in order to coordinate does not come naturally and easily to chimpanzees' [44]. This latter notion is also hard to reconcile with the rich evidence on highly flexible communicative strategies deployed for the solicitation and coordination of joint activities like consortship [66], social grooming [27,67,68] and social play [30,69–72].

Moreover, experimentally induced cooperative interactions might not deliver the same information as naturalistic and spontaneous social action coordination between conspecifics, and *vice versa*, because the decision-making is based on different motivational processes (‘food reward' versus ‘social reward'). The fact that apes do not coordinate or communicate habitually in experimental cooperation settings does not preclude that they would do so in their every day multimodal social interactions, the native context of their species-typical communication. The point here is not that experiments are irrelevant, but that research programmes might benefit from a more *inclusive* study design. This could entail a better integration of observational and experimental data and more ecologically valid experiments, fitting a species' natural behaviour (e.g. interruptions during spontaneous social grooming and conspecifics interacting with one another [27]; attention directed towards conspecifics' communicative signals or faces [73,74]) rather having humans interact with apes on species-atypical tasks [75]. A *combination* of both ecologically valid experiments and observations of spontaneous communication in joint activities of nonhuman great apes may deliver useful data to address these big debates.

**Figure 1.**
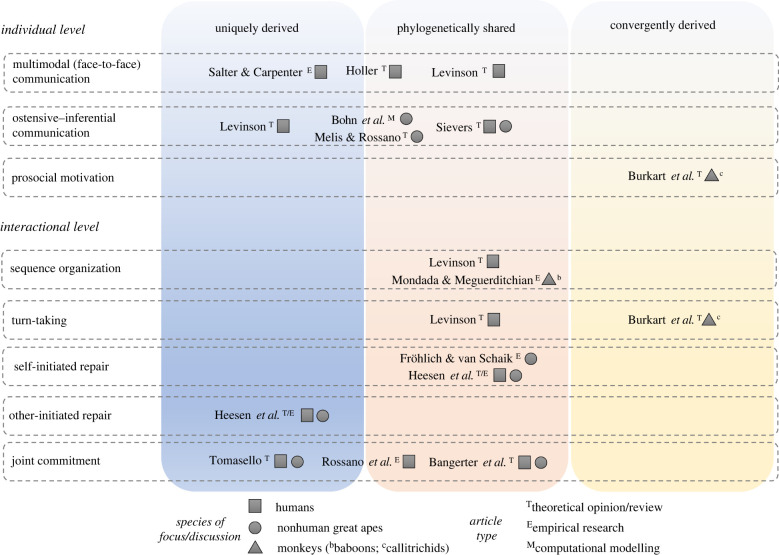
Overview of the contributions of this theme issue, indicating for each the covered interaction engine layer(s) and their discussed origins, study species and article type.

To dissect the interaction engine hypothesis, this theme issue has compiled a unique set of theoretical, computational modelling and empirical research articles (figure 1). In this synthesis, articles within and beyond the theme issue acknowledge that the human interaction engine is composed of a set of three layers with phylogenetically shared, convergently derived, and uniquely derived features. There appears to be a shared core, including features like multimodal face-to-face signalling [76], turn-taking [21,22,77,78], sequence organization [21,23,24,79], self-initiated repair [25,26,80,81] and some behavioural correlates linked to joint commitment [23,27–29].

As Burkart *et al*. [11] showed, specific affordances linked to a species' social system can favour the selection of cooperative behaviours also seen in the human interaction engine through convergence: callitrichid monkeys which engage in cooperative breeding also exhibit vocal turn-taking and a high level of prosociality. Turn-taking is thus an uncertain case, as it is has been discovered in the form of different communicative modalities and in primates that are not necessarily closely related, such as humans, different species of great apes, and callitrichid monkeys. For this reason, we acknowledge that specific human turn-taking capacities could be in part shared with the great apes [21,22,77,78] and in part convergently derived [11,82].

Other capacities, like ostensive–inferential communication, explicit joint commitment involving promises and agreements, and other-initiated repair, may represent derived traits in humans not shared with other animals (figure 1). Some of these, especially joint commitment and repair, probably have become more complex because of language [18], an issue that is open to further debate and goes beyond the scope of this issue. These capacities *in particular*, combined with the other shared and convergently derived interaction engine components, may have acted as stepping stones to conventional language. We acknowledge that this revised view of the human interaction engine is not carved in stone but represents a state-of-the-art perspective that will likely be adjusted with incoming future research. For instance, as noted by some authors, we still need further controls to consolidate evidence for certain components in great apes (e.g. joint commitment [44]).

## 4. Implications and outlook: what have we learned, and where to go from here?

In bringing together diverse contributions for this theme issue, our goal was to highlight the huge potential of social interaction as an object of study for human *and* nonhuman research, to clear up cloudy concepts for use in comparative research with animals, and to encourage further scientific discourse around social action coordination in nonhuman species. Through the integration of theoretical and empirical accounts on the coordination of communicative interactions in humans and nonhumans, this theme issue showed that direct scientific dialogue is essential in an interdisciplinary field heavily guided by loaded terminology rather than by species-agnostic empirical coding frameworks (e.g. [25,29,30]). Importantly, we do not suggest to lose focus of the study species and its socioecology, but to employ methods that deliver fair species comparisons (e.g. when studying a cognitive trait whose evolutionary origins are presumed to predate language, it would be inadequate to test it by applying the same experimental task to humans and a non-linguistic species if humans are allowed to speak [75]).

Importantly, our goal with the collection of these papers was neither to contest human-uniqueness claims, nor to merely point to the rather obvious lack of comparable data from nonhuman species. As Tomasello [44] notes in his contribution, through an interactive ‘pulling by the boosters' (i.e. contestants of human-uniqueness claims) and ‘pushing back by the scoffers' (i.e. defenders of human-uniqueness claims), the field of comparative science can reach an informed consensus on the big questions, one by one. It is also not a new point that features (or ‘layers') of the interaction engine are being added, or become more complex, rather than being entirely replaced in punctuated evolutionary processes (e.g. [6]), but we need more empirical evidence to better understand the order of emergence of these layers.

From our perspective as Guest Editors, five major aspects have become clear from this collection of expertise. First, we learned that social interaction can be scrutinized using an impressive variety of methodological approaches, including detailed video-based transcriptions of action sequences [24,25], classical ethological studies of naturalistic interactions [26,57], experimental paradigms [58], longitudinal research [43], and a computational modelling approach [64]. To our positive surprise, many of these conributions adopted a multimodal approach, bearing witness to long-awaited transitions toward a more holistic study of communicative interactions [41,83]. Moreover, several studies focusing on interaction engine features in nonhuman species were co-authored or even led by linguists or developmental psychologists who have built their careers at least partly on human communication (e.g. Mondada, Bohn and Dingemanse). This paints a promising picture of a fruitful cross-disciplinary dialogue emerging in this field.

Second, from looking at the synthetic summary of contributions in figure 1, it appears that the variability in interaction engine components across primates is a matter of *degree* rather than an all-or-nothing situation. What else was needed for language to evolve? Higher orders of intentionality and group-wide prosociality are now by many considered as the key ingredients of the human interaction engine that could have paved the way for the evolution of conventional languages, given that evidence for these features in nonhuman species remains scarce [3,44,58,64] and because these qualities are partly shared with other, cooperative breeding primates [11]. Nonetheless, it remains to be seen to what extent this disparity between nonhuman and human animals can be explained by methodological constraints. Additionally, figure 1 highlights research gaps on convergently derived features, which are important for understanding how and why social requirements linked to cooperative breeding can actually foster the evolution of such capacities in species engaging in such a breeding system (humans and callitrichids, but not in apes) [11].

Third, we learned about important modulators of communicative performance [26,58,64]. For example, studies showed that the relationship between interactants (e.g. social tolerance as predicted by the kin and dominance relationship) is an important determinant of communicative production and comprehension, irrespective of research setting (wild, captive) and research design (observational, experimental).

Fourth, in returning to the question of which selective pressures might have acted upon early communication systems in favour of language evolution, we realize that many debates are still unsolved. We still do not know which unique elements of the interaction engine are in fact consequences of language rather than have prepared for it. Was it the need for cooperation in stag hunt scenarios that favoured language emergence, or were high levels of cooperation only possible because of language use [58]? Indeed, some cognitive performances are possibly facilitated by language, such as future planning or negotiations of commitments [18]. Also, although self-initiated repair seems to be present in nonhuman great apes [25,26], other-initiated repair may only be possible through language, as it involves conventional repair cues and sophisticated articulation of miscomprehension [18,84]. Because language transformed intersubjectivity in such unique ways, some scholars might argue that the social settings in which modern humans interact are not at all comparable with those of other species. Yet, as Sievers argues, we should not shy away from empirically assessing a certain concept just because a previous theory has *a priori* assumptions of the cognitive abilities driving it [34].

Last, it remains unclear how different interaction engine ingredients operate across interaction contexts. For instance, does the prevalence of certain interaction engine features vary between interactions characterized by asymmetry (coordination of two distinct roles, e.g. in consortship) versus those characterized by symmetry (coordination of reversible roles, e.g. in social grooming or play)? One would assume that among dyads with predictable interaction outcomes (e.g. dyad with high rank difference, mother–offspring dyad) the extent of negotiations and role-reversals is limited, hence featuring less coordination, compared with interactions with unpredictable outcomes (e.g. partners equal in rank, unfamiliar partners).

Overall, this theme issue has backed up the idea that humans' capacity for joint action is built on shared, uniquely and convergently derived interactional abilities [35]. Through the power of the language faculty, human sociality has then transformed in significant ways [18]. All contributors of this issue have tried to enhance our understanding of which kinds of capacities might have preceded and favoured the evolution of language, including those who employed comparative and developmental approaches. The various contributions also illustrated the diversity of methods by which social interaction and communication can be studied, both within and beyond our own species. Comparative research has just started to engage in a truly cross-disciplinary exchange, opening new windows for promising future projects. As a main impact, we hope this issue will further solidify and establish the place of comparative research on social interaction in the behavioural sciences, and spur further research on interaction engine properties in nonhuman species.

## Data Availability

This article has no additional data.
